# DNA methylation as a tool to explore ageing in wild roe deer populations

**DOI:** 10.1111/1755-0998.13533

**Published:** 2021-10-30

**Authors:** Jean‐François Lemaître, Benjamin Rey, Jean‐Michel Gaillard, Corinne Régis, Emmanuelle Gilot‐Fromont, François Débias, Jeanne Duhayer, Sylvia Pardonnet, Maryline Pellerin, Amin Haghani, Joseph A. Zoller, Caesar Z. Li, Steve Horvath

**Affiliations:** ^1^ Laboratoire de Biométrie et Biologie Evolutive UMR5558 Université de Lyon Université Lyon 1 CNRS Villeurbanne France; ^2^ Université de Lyon VetAgro Sup Marcy‐l'Etoile France; ^3^ Direction de la Recherche et de l'Appui Scientifique Office Français de la Biodiversité Unité Ongulés Sauvages Gap France; ^4^ Human Genetics David Geffen School of Medicine University of California Los Angeles California USA; ^5^ Department of Biostatistics Fielding School of Public Health University of California Los Angeles California USA

**Keywords:** epigenetic clock, growth, life‐history, senescence, sex differences in lifespan

## Abstract

DNA methylation‐based biomarkers of ageing (epigenetic clocks) promise to lead to new insights into evolutionary biology of ageing. Relatively little is known about how the natural environment affects epigenetic ageing effects in wild species. In this study, we took advantage of a unique long‐term (>40 years) longitudinal monitoring of individual roe deer (*Capreolus capreolus*) living in two wild populations (Chizé and Trois‐Fontaines, France) facing different ecological contexts, to investigate the relationship between chronological age and levels of DNA methylation (DNAm). We generated novel DNA methylation data from *n* = 94 blood samples, from which we extracted leucocyte DNA, using a custom methylation array (HorvathMammalMethylChip40). We present three DNA methylation‐based estimators of age (DNAm or epigenetic age), which were trained in males, females, and both sexes combined. We investigated how sex differences influenced the relationship between DNAm age and chronological age using sex‐specific epigenetic clocks. Our results highlight that old females may display a lower degree of biological ageing than males. Further, we identify the main sites of epigenetic alteration that have distinct ageing patterns between the two sexes. These findings open the door to promising avenues of research at the crossroads of evolutionary biology and biogerontology.

## 1 INTRODUCTION

The last decades have seen a growing interest into the study of ageing in the wild (Fletcher & Selman, 2015; Gaillard & Lemaître, 2020; Monaghan et al., 2008). The starting point of this increase is undeniably the compilation of evidence reporting that ‐ in populations of animals in the wild ‐ senescence occurs in demographic performance (actuarial senescence: Brunet‐Rossinni & Austad, 2006; Nussey et al., 2013; reproductive senescence: Lemaître & Gaillard, 2017; Nussey et al., 2013), phenotypic performance (e.g., body mass: Douhard et al., 2017; Nussey et al., 2011; foraging efficiency: Lecomte et al., 2010; MacNulty et al., 2009) and physiological traits (e.g., immune parameters, Nussey et al., 2012; haematological parameters, Jégo et al., 2014; steroid levels, Sugianto et al., 2020). Nowadays, the age‐specific decline in demographic and physiological performance is considered to be the rule rather than the exception in the wild, at least in mammals and birds (Gaillard & Lemaître, 2020; Nussey et al., 2013; see also Zajitschek et al., 2020 in insects).

Animal populations in which individuals are monitored from birth to death in the wild provide a unique (but largely untapped) resource for studying individual differences in health and mortality risk at old ages (Gaillard & Lemaître, 2020; Lemaître, Pavard, et al., 2020). Multiple lines of evidence emphasize the relevance of such longitudinal and individually‐based data. First, the vast majority of the current research in biogerontology has focused on inbred laboratory organisms with no or low genetic variation and maintained under controlled conditions (e.g., *Caenorhabditis elegans*, *Drosophila melanogaster*, laboratory rodents, Partridge, 2010). Studies performed on those species have led to major breakthroughs in the mechanisms regulating the ageing process from the molecular to the individual level (Kennedy et al., 2014; López‐Otín et al., 2013). However, their findings can be difficult to extrapolate to species living in more complex environments (Briga & Verhulst, 2015), with diverse genetic background and much longer lifespan and thereby different life history strategies, such as humans (Perlman, 2016). Even if ageing studies of non‐human primates kept in captive conditions are increasing (Jasinska, 2020; Languille et al., 2012), the full diversity of mammalian species displaying life‐history traits and life styles similar to the ones observed in humans (e.g., whether there are socially monogamous, long‐lived, provide extensive periods of parental care or create tight social bonds with conspecifics) needs to be embraced and generalized. In addition, the study of the ageing process in the wild enables ‐ by essence ‐ to investigate the role played by the environment, an important piece of the ageing conundrum. Similar to what has been described in human populations (e.g., Robine et al., 2012 in the context of climatic variables), there is increasing evidence that environmental factors modulate ageing patterns in animal populations (Holand et al., 2016; Nussey et al., 2007). For instance, it is increasingly recognized that the social environment can have a major influence on health and mortality risk at late ages (Berger et al., 2018; Snyder‐Mackler et al., 2020), by notably interacting with some hallmarks of ageing (e.g., telomere dynamics in Seychelle warblers, *Acrocephalus sechellensis*, Hammers et al., 2019). In addition, while mammalian females generally live longer than males in the wild (Lemaître, Ronget, et al., 2020), as commonly observed in humans or laboratory rodents (Austad & Fischer, 2016; Zarulli et al., 2018), the exact mechanisms modulating these sex differences in survival are yet to be deciphered (Marais et al., 2018; Tower, 2017). In that context, the focus on wild populations can be particularly relevant as the magnitude of sex differences in lifespan is probably modulated by environmental conditions, in interaction with the sex differences in genetic background (Lemaître, Ronget, et al., 2020; Tidière et al., 2020). Finally, widening the scope of model species for ageing research can provide important insights for “healthspan extension”, notably by targeting wild animal populations displaying extended lifespan compared to the one expected for their body size (Austad, 2010) and by including more appropriate senescence metrics (Lemaître, Garratt, et al., 2020; Ronget & Gaillard, 2020). To reach these goals, accurate markers of both chronological and biological ages on a wide range of organisms are required. In that context, the pan tissue epigenetic clock based on DNA methylation (see Horvath, 2013) constitutes a promising tool for the study of ageing in wild populations of non‐model organisms (Lu et al., 2021; Parrott & Bertucci, 2019; Wilkinson et al., 2021).

DNA methylation (DNAm) of cytosine residues within CpG dinucleotides (5‐methyl‐cytosine) across the genome constitutes a key epigenetic DNA modification tightly linked to the ageing process (Horvath & Raj, 2018). Indeed, DNA methylation patterns accurately predict chronological age in humans (Horvath, 2013; Jung & Pfeifer, 2015) but also in multiple other species (Lu et al., 2021; Paoli‐Iseppi et al., 2017; Wilkinson et al., 2021). Such strong relationship between chronological age and DNAm has been found in many cell types (e.g., white blood cells, brain, liver; see Horvath, 2013). A comparative analysis of methylomes indicates that methylation can also be used to assess reliably physiological ageing across mammals (Wang et al., 2020). The discrepancy between epigenetic age and chronological age (i.e., epigenetic acceleration) is associated in humans with a wide range of metabolic, infectious and degenerative diseases (Horvath et al., 2014; Horvath & Levine, 2015), as well as cancer (Levine et al., 2015) and mortality (Chen et al., 2016; Christiansen et al., 2016; Marioni et al., 2015). We hypothesize that DNA methylation profiles integrates environmental effects that might modulate the pace of the epigenetic clock. To address this hypothesis, we studied epigenetic ageing in the wild and in a sex‐specific way.

In this study, we took advantage of a unique long‐term (>40 years) longitudinal monitoring of individual roe deer (*Capreolus capreolus*) living in two populations facing different ecological contexts to investigate the relationship between chronological age and levels of DNA methylation. All roe deer used in this study have been captured within their first year of life, when age can be accurately assigned (Hewison et al., 1999). First, we expected that the epigenetic clock built from peripheral blood leucocyte DNA would provide an accurate estimation of the chronological age of the roe deer in the wild. Second, we performed an epigenome wide association analysis (EWAS) to identify CpGs that were the most likely to be associated with ageing in roe deer. Third, since male roe deer show both higher adult mortality and rate of actuarial senescence than females (Gaillard et al., 2004), we tested the hypothesis that males should have an epigenetic acceleration higher than females. Moreover, thanks to the epigenome wide association analysis, we expected to identify specific CpGs displaying sex‐specific DNAm ageing profiles.

## 2 MATERIALS AND METHODS

### 2.1 Study populations

We sampled roe deer living in two enclosed forests with markedly different environmental contexts: Trois‐Fontaines (TF) and Chizé (CH). The Trois‐Fontaines forest (1360 ha) is located in north‐eastern France (48°43'N, 4°55'E) and is characterized by a continental climate, moderately severe winters and warm and rainy summers. This site has rich soils and provides high quality habitat for roe deer (Pettorelli et al., 2006). In contrast, the Chizé forest (2614 ha) is located in western France (46°50'N, 0°25'W) and is characterized by temperate oceanic climate with Mediterranean influences. This site has a low productivity due to poor quality soils and frequent summer droughts (Pettorelli et al., 2006), and thereby provides a quite poor habitat for roe deer in most years. Individuals from these two populations have been intensively monitored using a long‐term Capture‐Mark‐Recapture programme since 1975 and 1977 (for Trois‐Fontaines and Chizé, respectively). At each site, 10–12 days of capture using drive‐netting are organized every year between December and March (see Gaillard et al., 1993 for details on capture sessions), which allows capturing and measuring about half of the population every year. Once a roe deer is captured, its sex and body mass (to the nearest 50 g) are recorded and a basic clinical examination is performed. All individuals included in our analyses were of known age because they were either caught as newborn in spring (see Delorme et al., 1988 for further details) or as ca. 8 months old during winter captures, when they still have their milk teeth (most often incisors and always premolars, Flerov, 1952). Individuals are equipped with individually recognizable ear‐tags and collars (either numbered, VHF or GPS collars), get a unique id, and are closely monitored throughout their lifetime using subsequent winter captures and observations in the field.

### 2.2 Roe deer blood samples and DNA extraction

In 2016 and 2017, we collected blood samples (up to 1 ml per kg of body mass) into heparin‐lithium collection tubes (4 ml Vacutainer, BD Medical) from the jugular vein. Within 30 min of sampling, the blood was centrifuged at 3000 *g* for 10 min and the plasma layer was removed before washing the cells with an equivalent volume of 0.9% w/v NaCl solution. After a second centrifugation, the intermediate buffy coat layer, comprising mainly leukocytes, was collected in a 1.5‐ml Eppendorf tube and immediately frozen at −80°C in a portable freezer (Telstar SF 8025) until further use.

We extracted genomic DNA from leucocytes using the Macherey‐Nagel NucleoSpin Blood QuickPure kit. DNA purity was assessed using a Nanodrop ND‐1000 spectrophotometer (Thermo Scientific). For all samples, the purity absorption range was 1.7–2.0 for the 260/280 nm ratio and >1.8 for the 260/230 nm ratio. We selected 96 samples by balancing the numbers of individuals among ages, and between populations and sexes. DNA concentration was determined spectrophotometrically using the Qubit assay kit. DNA samples were then diluted in ultrapure water to reach a concentration of ~70 ng.µl^−1^ and displayed in a microplate to complete the DNA methylation protocol (see below).

### 2.3 DNA methylation data

We generated DNA methylation data using the custom Illumina chip "HorvathMammalMethylChip40". The mammalian methylation array is attractive because it provides very high coverage of highly conserved CpGs in mammals (Arneson et al., 2021). Each probe is designed to cover a certain subset of species, such that overall all species have a high number of probes (Arneson et al., 2021). The particular subset of species for each probe is provided in the chip manifest file can be found at Gene Expression Omnibus (GEO) at NCBI as platform GPL28271 and on the GitHub page reported in our section on data availability.

The SeSaMe normalization method was used to define beta values (methylation levels) for each CpG (Zhou et al., 2018). The SeSaMe normalization applies to one sample at a time.

### 2.4 Quality control and final data set

We implemented several steps of quality control. First, we measured DNA quality (see above). Second, we carried out unsupervised hierarchical clustering (based on the inter‐array correlation) to find putative outliers. This analysis revealed one severe outlier (Figure S1) which was subsequently removed from the analysis. Third, we developed a random forest‐based predictor of sex to find putative labelling errors. According to the out‐of‐bag predictions of sex, one of the male samples actually derived from a female. To err on the side of caution, we removed this sample from our analysis. After omitting the sample, the out‐of‐bag estimate of accuracy was 100%.

The 94 roe deer samples (i.e., 45 male samples, 44 female samples) analysed in this study correspond to 83 individuals (i.e., 11 individuals were sampled both in 2016 and 2017) aged from 8 months to 13.5 years of age. This age range encompasses most of the roe deer lifespan as individuals older than 15 years of age are rarely observed in the wild (the oldest age ever recorded for a roe deer monitored in the wild being 17.5 years old, Gaillard et al., 1998).

### 2.5 Epigenetic clock construction

Statistical details (including CpGs and coefficient values) are presented in the Supporting Information. Our epigenetic clocks for roe deer were trained on 29,846 CpGs (covariates) that could be mapped to the Ovir.te_1.0 genome assembly. Epigenetic clocks are multivariate regression models whose dependent variable (chronological age) was regressed on the CpGs (covariates) using an elastic‐net regression model (Friedman et al., 2010). Toward this end, we used the R package glmnet (version v.4.0‐2) with argument alpha =.5. To estimate the lambda parameter in each training set, we used tenfold cross validation. We used two strategies to arrive at unbiased estimates of accuracy: (1) leave‐one‐out cross validation (LOO) and (2) data splitting (e.g., when training clocks in one group and applying it to another group). LOO involved iteratively training clocks in n‐1 samples and applying it to the left out sample. The final deer clocks (presented in the Supporting Information) were trained on all *N* = 94 samples.

To further control for nonindependence, we also estimated the DNAm age in roe deer based on Universal Mammalian clock (“Universal log‐linear transformed age” clock, see Lu et al., 2021 for details). We used the tenfold cross validation estimates of the universal clock to ensure an unbiased analysis.

### 2.6 Statistical analyses

We first aimed to detect the function providing the best fit of the relationship linking DNAm age and chronological age. We thus compared three models corresponding to (1) an absence of relationship (constant model), (2) a constant increase of DNAm with age (linear model), and (3) a nonlinear increase of DNAm with age (quadratic model, Table S1). The most parsimonious model was selected using the Akaike Information Criterion (AIC). We calculated AIC weights (AICw) to assess the relative likelihood that a given model was the best among the three fitted models (Burnham & Anderson, 2002). We selected the model with the lowest AIC, but when the difference in AIC (denoted ΔAIC) between two competing models was less than two units, we retained the simplest model in accordance with parsimony rules (Burnham & Anderson, 2002). We used a strictly similar approach to analyse the relationship linking DNAm age estimated with the Universal Mammalian Clock and chronological age.

Second, we analysed factors that could explain the between‐sample variation in the epigenetic acceleration. Following Horvath (2013), we computed the epigenetic acceleration as the residuals of the models that best described the relationship between DNAm age and chronological age (i.e., quadratic model, see below). We then investigated whether the epigenetic acceleration was influenced by sex, population and roe deer body mass (measured at capture) for the three main life stages in terms of survivorship in roe deer (Gaillard et al., 1993): juvenile (<1 year of age), prime‐age (1–8 years of age), and senescent (>8 years of age). As roe deer lived on average longer in the population facing a marked food limitation than in the population living in a more productive forest, probably in relation to weaker tooth wear (Veiberg et al., 2007), we expected the epigenetic acceleration to be higher in the latter. For both prime‐age and senescent life stages we ran a set of 14 models with the epigenetic acceleration as the dependent variable and sex, population and body mass as the independent variables (see Table S2 for a full list of models). To avoid fitting over‐parameterized models, we did not include any three‐way interactions. Due to the low number of individuals of 1 year of age (*N* = 8), we only fitted four models for the juvenile life stage, the constant model (i.e., no detectable influence of any independent variable) and models including either a linear effect of body mass, sex or population. Given that roe deer reach about two‐third of their final body mass by 8 months of age (Hewison et al., 2011), body mass at 8 months of age reliably measures the overall allocation to growth by a roe deer, allowing us to explore whether a high allocation of resources towards growth can be associated with long‐term ageing costs (a long‐standing question in evolutionary biology of ageing, for example, Metcalfe & Monaghan, 2003), detectable through an epigenetic acceleration. In all cases, the best fitting model was selected using AIC (see above). AIC tables are provided in Supporting Information.

Third, we investigated further how sex influenced the relationship between DNAm age and chronological age through the use of sex‐specific epigenetic clocks. For this purpose, we first built an epigenetic clock (called “female clock”) using data from female samples only, and then investigated the relationship between the female clock and both male and female chronological age. We performed a similar analysis with an epigenetic clock built using data from male samples only (called “male clock”). In our study, 11 individuals were sampled both in 2016 and 2017. Thus, to account for this pseudoreplication problem (sensu Hurlbert, 1984) we replicated all analyses using linear mixed‐effects models, which included a random effect of individual roe deer, using the R‐package lme4 (Bates et al., 2015). For all models, results were qualitatively unchanged (see Tables S3 and S4) and for the sake of simplicity, we only report results from simple linear models below.

Fourth, we performed an epigenome wide association (EWAS) study of chronological age in roe deer. Unfortunately, a complete genome assembly is not available for roe deer. Therefore, we performed an EWAS analysis based on the related White‐tailed deer, *Odocoileus virginianus* (Ovir.te_1.0 genome assembly). In total, 29,846 CpGs from the HorvathMammalMethylChip40 were aligned to loci that are proximal to 6314 genes in the Ovir.te_1.0 genome assembly. Due to the high interspecies conservation of the probes on the array, findings can be extrapolated to roe deer, and even humans or other mammalian species. To assess the potential mechanism, we used a multivariate regression model to identify the CpGs that have a distinct pattern of DNAm ageing between the sexes. We used two multivariate models. In the first model, DNAm levels of an individual CpGs were regressed on sex‐specific chronological age to identify the loci with DNAm ageing that are shared between sexes (“Age” main effect), and also the basal sex difference that is independent of chronological age (“Sex” main effect). In the second model, we included an interaction term to identify the CpGs with distinct DNAm ageing between males and females.

For all EWAS results, the top 1000 CpGs (up to 500 per direction) were used for gene set enrichment analysis. We used the rGREAT package for the analysis (McLean et al., 2010), with a 50 kb window for assigning the CpGs to the adjacent genes. The background included human Hg19 limited to 29846 probes mapped to Ovir.te_1.0 genome.

## 3 RESULTS

### 3.1 Relationship between DNAm and chronological age

**FIGURE 1:**
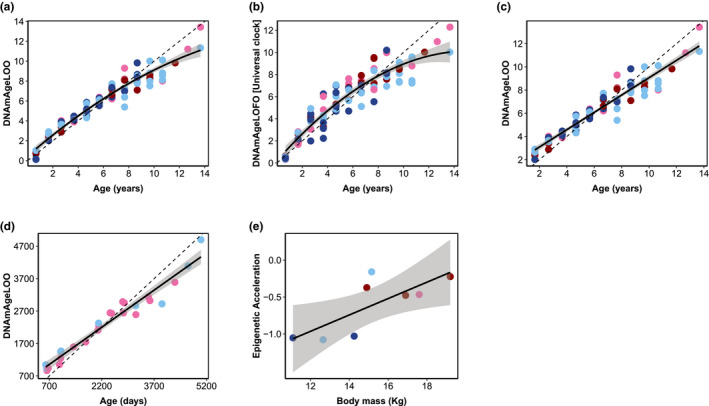
Epigenetic clock for roe deer built with individual methylation profiles from white blood cells DNA. (a) Epigenetic clock for known‐aged individuals between 8 months and 14 years of age (*N* = 94; R^2^ = 0.94); (b) Relationship between DNAm Age and chronological age in roe deer based on the universal mammalian clock (*N* = 94; R^2^ = 0.87). (c) Epigenetic clock for adult roe deer (i.e. >1 year old, *N* = 86; R^2^ = 0.92); (d) Epigenetic clock for individuals where the exact age in days was known (*N* = 26; R^2^ = 0.95); (e) Relationship between the epigenetic acceleration and the body mass for juveniles (i.e., 8 months, *N* = 8). The epigenetic age (DNAmAgeLoo) is expressed in years. In all graphs, the dashed line corresponds to the regression line y = x. Males in Chizé are in dark blue, females in Chizé are in light blue, males in Trois‐Fontaines are in dark red and females in Trois‐Fontaines are in light red

**FIGURE 2:**
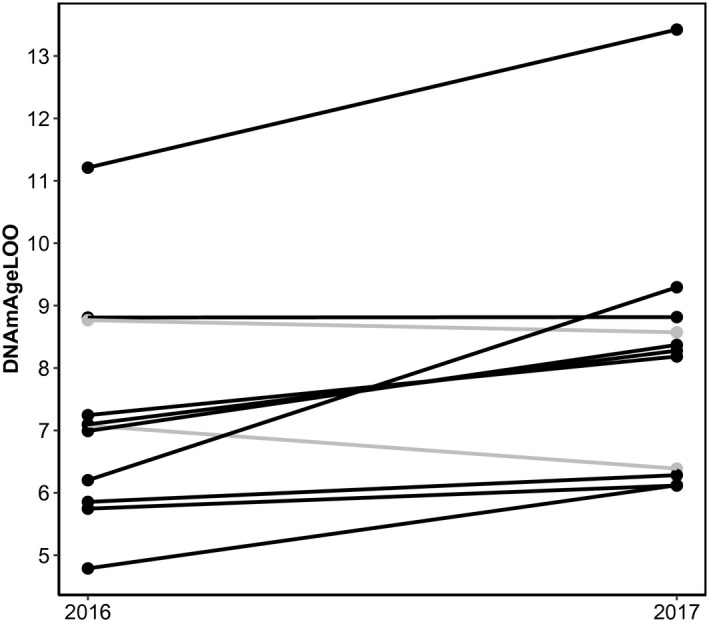
Within‐individual change in DNAmAgeLoo across the two consecutive years of our study (2016 and 2017) (*N* = 11). DNAm age increases in nine out of 11 individuals between the two years of the study

The model that best described the relationship between DNAm and chronological age was the quadratic model (Table 1a; Figure 1a; Table S1). A similar pattern was also observed when DNAm Age was estimated with the Universal Mammalian Clock (Figure 1b; Table 1b). This relationship may be due to the fact that DNA methylation accumulates at a faster rate during the growth and development of juveniles than later in life when roe deer have reached their full size (at 2 years of age, roe deer have reached >90% of their asymptotic body mass, Hewison et al., 2011). Accordingly, the model that best described the relationship between DNAm and chronological age in adults only (2–14 years of age) was the linear model (slope of 0.75 ± 0.02, *N* = 86, R^2^ = 0.92; Figure 1c; Table S1). For a subset of 26 adults from the Trois‐Fontaines population, the exact date of birth was known meaning that it was possible to compute age at a very fine‐scale resolution (i.e., in days). The use of this chronological age data measured in days slightly improved the fit of the relationship compared to the one obtained with the chronological age measured in years but the slope was left unchanged (slope of 0.76 ± 0.04, *N* = 26, R^2^ = 0.95; Figure 1d). As expected, in the vast majority (i.e., 81.8%, nine out of 11 individuals) of the individuals for which we estimated the epigenetic age in both 2016 and 2017, we observed an increase in DNAm age (Figure 2).

**TABLE 1:** Parameters of the selected models in the main text

Dependent variables		Estimate	SE	t
(a) DNAm age	Intercept	0.46	0.22	2.084
	Age	1.09	0.08	13.98***
	Age^2^	–0.02	0.006	–3.86***
(b) DNAm age	Intercept	0.2	0.32	0.645
	Age	1.29	0.11	11.57***
	Age^2^	–0.04	0.009	–4.87***

Note

(a) Quadratic model describing the relationship between DNAm age estimated with the roe deer clock and chronological age (*N* = 94; R^2^ = 0.94). (b) Quadratic model describing the relationship between DNAm age estimated with the Universal Mammalian Clock and chronological age in roe deer (*N* = 94; R^2^ = 0.87).

*** *p* < .001.

### 3.2 Epigenome wide association study of chronological age

**FIGURE 3:**
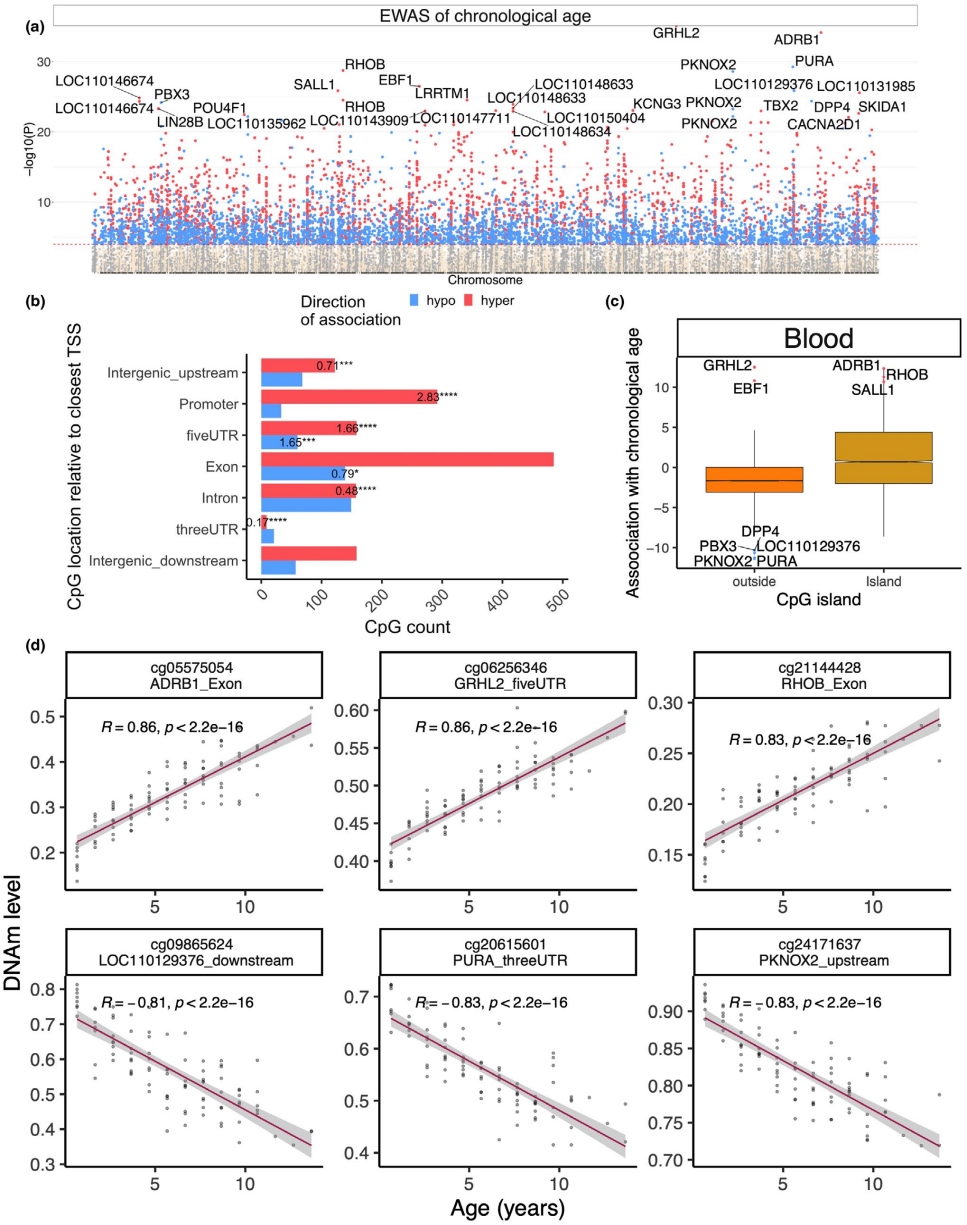
Epigenome‐wide association (EWAS) of chronological age in the blood of roe deer. (a) Manhattan plot of the EWAS of chronological age. Since the genome assembly is not available for roe deer, the coordinates are estimated based on the alignment of Mammalian array probes to White‐tailed deer (Ovir.te_1.0) genome assembly, a related species to roe deer. The direction of associations with *p* < 10^−8^ (red dotted line) is highlighted by red (hypermethylated) and blue (hypomethylated) colours. The top 30 CpGs were labeled by the neighboring genes. (b) Location of top CpGs in each tissue relative to the closest transcriptional start site. Top CpGs were selected at *p* < 10^−8^ and further filtering based on z score of association with chronological age for up to 500 in a positive or negative direction. The labels indicate the odds ratio of regions with a statistically significant proportion change than the background. Fisher's exact test *p*‐values are reported as: ****p < 10^−4^, ***p < 10^−3^, **p < 10^−2^, * *p* < .05. (c) CpG islands have a higher positive association with age (hypermethylation) than other sites. (d) Scatter plot of the top CpGs associated with age in roe deer

**FIGURE 4:**
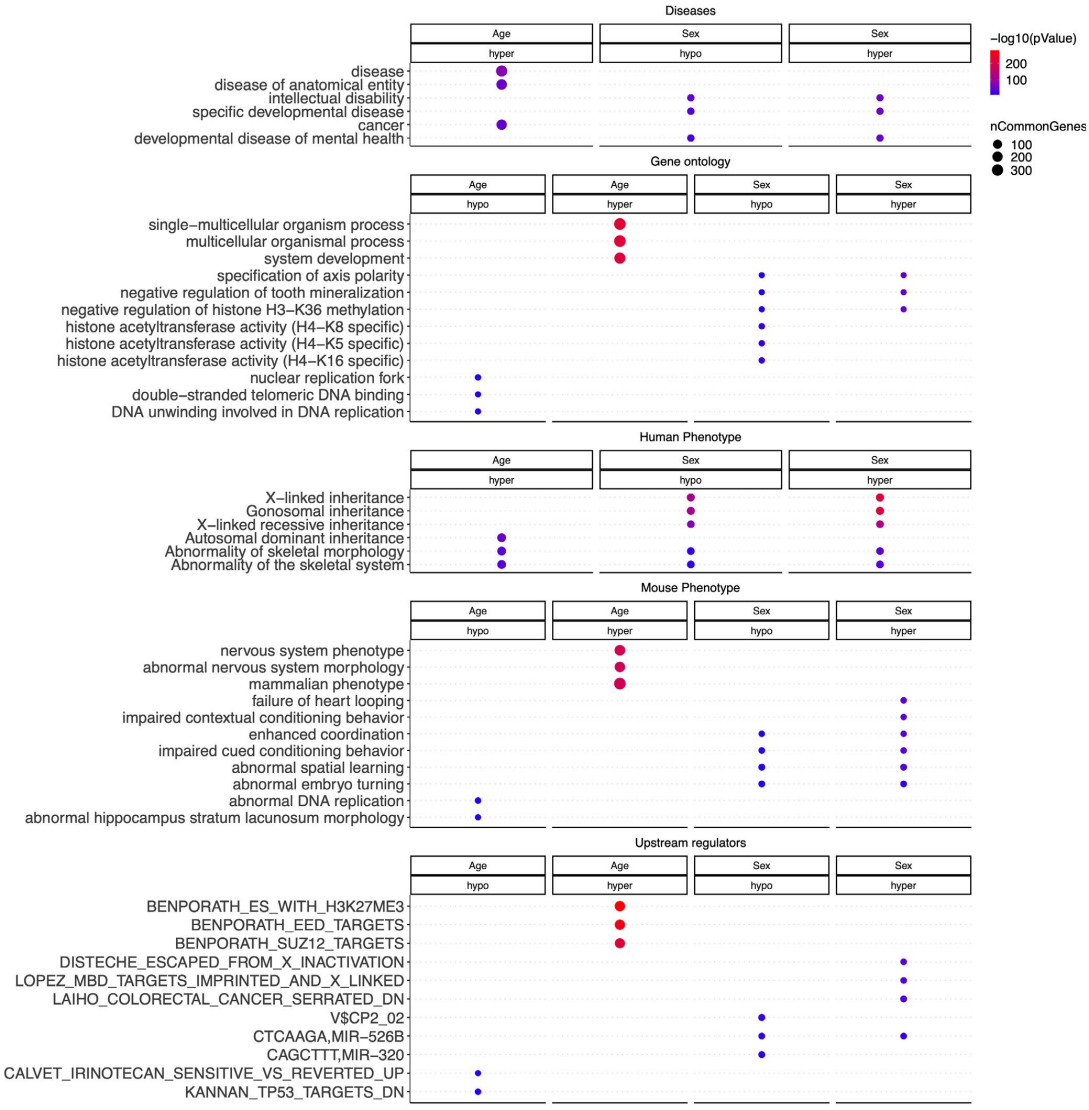
Functional enrichment analysis of EWAS of age and sex. The analysis was done using the genomic region of enrichment annotation tool (McLean et al., 2010). The gene‐level enrichment was done using GREAT analysis (McLean et al., 2010) and human Hg19 background. The top three enriched data sets from each category (Canonical pathways, diseases, gene ontology, human and mouse phenotypes, and upstream regulators) were selected and further filtered for significance at *p* < 10^−4^

Our EWAS of age used the R function "standardScreeningNumericTrait" in the WGCNA R package (Langfelder & Horvath, 2008). This R function correlates each CpG with the numeric phenotype (age) and outputs a Student *t* test statistic referred to as (Z statistic) and a corresponding *p*‐value. The EWAS results revealed that chronological age has a profound effect on DNAm levels (Figure 3). Even at the arguably overly stringent *p*‐value threshold of *p* < 10^−8^ (corresponding to Bonferroni correction for 5 million independent hypothesis tests), 1908 (out of 29,846) CpGs exhibited a significant correlation with age. The most significant CpGs were located in *GRHL2* 5'UTR (z = 12.5), *ADRB1* exon (z = 12.3), and *PURA* 3'UTR (z = –11.4) (Figure 4a). Ageing‐associated CpGs in roe deer leucocyte DNA were distributed in all genic and intergenic regions that can be defined relative to transcriptional start sites (Figure 3b). However, promoter (*p* = 7.1e‐44) and 5'UTR (*p* = 4e‐8) regions contained a significant number of CpGs that gained methylation levels with age (Figure 3b). The same can be observed for CpGs located in CpG islands (Figure 3c).

Gene level enrichment analysis of the significant CpGs highlighted changes in development, nervous system, O‐Glycan metabolism, cancer, and immune system, all of which are associated with ageing biology in humans and other species (Figure 4). The analysis suggested that CpGs that gain methylation with age are located in regions marked by H3K27me3 and are targeted by polycomb repressive complex 2 proteins such as EED. EED is associated with H3K27Me3 marks and is a member of the multimeric Polycomb family protein complex 2 that maintains the transcriptional repressive states of genes, and plays a role in the regulation of several cell states including cellular senescence (Ito et al., 2018).

### 3.3 Factors affecting the epigenetic acceleration

When focusing on juveniles only, the epigenetic acceleration was best explained by body mass (Table S2) as shown by the positive relationship between these two variables (slope of 0.11 ± 0.04, R^2^ = 0.58; *N* = 8, Figure 1e). On the contrary, for adults, the constant model of the epigenetic acceleration was selected. We then investigated in more details the factors potentially explaining the epigenetic acceleration in adults by running separate analyses for prime‐age and senescent adults. However, in both cases, the constant model was retained (Table S2).

### 3.4 Influence of sex on the relationship between DNAm age and chronological age in adults

**FIGURE 5:**
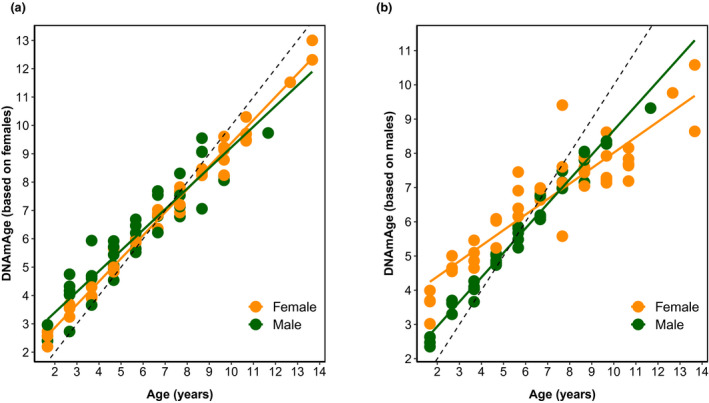
Sex‐specific epigenetic clock in roe deer. (a) Relationship between DNAm age estimated with the female clock and both male and female chronological ages (b) Relationship between DNAm age estimated with the male clock and both male and female chronological ages. In all graphs, the dashed line corresponds to the regression line y = x. Females are displayed in orange and males in green

When using the female clock, an interactive effect of age and sex occurred (Table 2a, Table S4). As expected, the fit was much better in females (slope of 0.81 ± 0.01, *N* = 46, R^2^ = 0.99) than in males (slope of 0.73 ± 0.04, *N* = 40, R^2^ = 0.87). Interestingly, most male's DNAm located above the line where DNAm age exactly matched chronological age, meaning that males are biologically older than their chronological age estimated using the female clock (Figure 5a). The opposite pattern occurred when using the male clock (Table 2b; Table S4; Figure 5b). Beyond 7 years of age, females were consistently biologically younger than their chronological age estimated from the male clock (Figure 5b). As expected, the fit was also much better for males (slope of 0.72 ± 0.02, *N* = 40, R^2^ = 0.98) than for females (slope of 0.46 ± 0.03, *N* = 46, R^2^ = 0.83) when using the male clock.

**TABLE 2:** Parameters of the models testing for an interaction between chronological age and sex using (a) Parameters obtained with the female clock (*N* = 94; R^2^ = 0.95) (b) Parameters obtained with the male clock (*N* = 94; R^2^ = 0.92)

Dependent variables		Estimate	SE	t
(a) DNAm age (female epigenetic clock)	Intercept	1.23	0.18	6.80***
	Age	0.81	0.02	34.35***
	Sex	0.7	0.27	2.58*
	Age * Sex	–0.09	0.04	–2.08*
(b) DNAm age (male epigenetic clock)	Intercept	3.49	0.19	18.60***
	Age	0.46	0.02	18.49***
	Sex	–1.98	0.28	–6.98***
	Age * Sex	0.26	0.04	6.17***

* *p* < .05.

*** *p* < .001.

### 3.5 CpGs whose ageing patterns differ by sex

**FIGURE 6:**
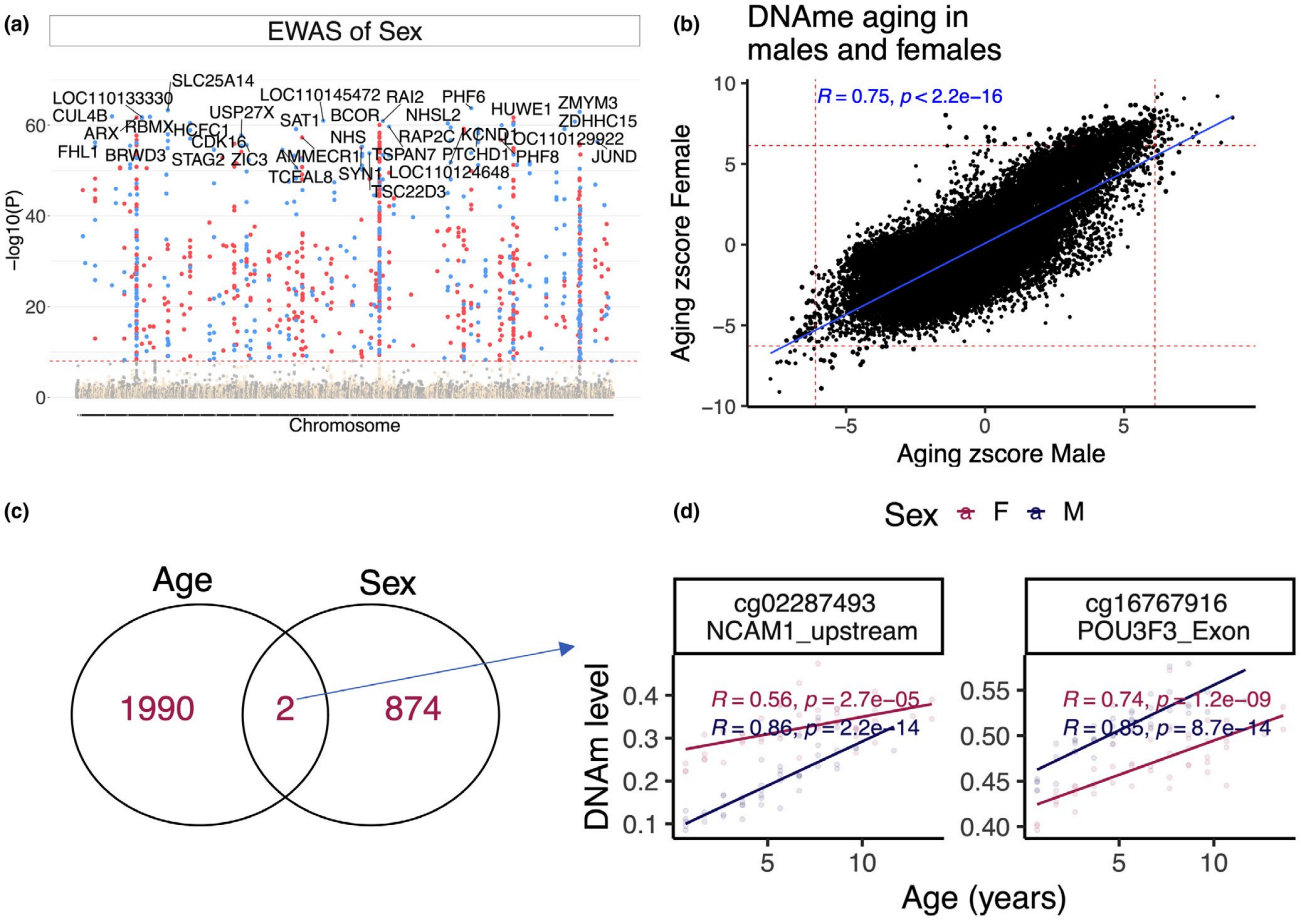
DNA methylome of sex differences in roe deer. (a) Manhattan plots of basal sex differences. The male is the reference variable to estimate the direction of change. Sample sizes: Males, 45; Females, 49. The coordinates are estimated based on the alignment of Mammalian array probes to White tailed deer (Ovir.te_1.0) genome assembly. The red line in the Manhattan plot indicates *p* < 1e^−8^. (b) Scatter plots DNAm aging between male and female roe deer. This is based on a stratified analysis of EWAS of age for each sexes. (c) Venn diagram of the overlap of sex related CpGs with EWAS of age. (d) Scatter plot of the CpGs with basal sex differences that also change with age

At genome‐wide significance (*p* < 10^−8^), a total of 876 CpGs were differentially methylated between males and females (Figure 6a). Methylated CpGs (DMCs) differentially associated with age between males and females were biased in specific scaffold chromosomes, which are expected to be the homologs of sex chromosomes in humans and other mammals. Functional analysis of these CpGs suggested differences in intellect, nervous system development, histone modifications, and X‐linked inheritance.

Since the epigenetic acceleration was faster in males, we screened for differences in ageing pattern between sexes. Notably, DNAm ageing was highly correlated (r = .75) between male and female roe deer (Figure 6b). To further explore the potential sex difference, we performed an EWAS of sex (adjusted for age as covariate) to identify the CpGs with basal (mean methylation) differences between sexes. We have identified two CpGs with basal difference between sexes that also related to age roe deer (Figure 6c): NCAM1 upstream, and POU3F3 exon (Figure 6d). The basal difference in these CpGs could underlie accelerated epigenetic ageing in males. For example, the loci in POU3F3 exon have higher baseline and ageing rate in males than females. POU3F3 (POU class 3 homeobox 3) is located on human chromosome 2 and involved in neuronal development.

## 4 DISCUSSION

Our findings highlight a very tight correlation between epigenetic and chronological age in two populations of roe deer intensively monitored in the wild. The quality of the fit of the selected model describing the relationship between epigenetic and chronologic age over the roe deer entire lifespan was particularly high (R^2^ of 0.94; Median absolute difference: 0.558 years), which adds to the increasing evidence that the epigenetic tool constitutes an accurate method to estimate age in vertebrate populations in the wild (Paoli‐Iseppi et al., 2017). So far, a wide range of techniques based on tooth wear are generally used to assign age in wild mammals (Morris, 1972, Pérez‐Barbería et al., 2014 for a review in red deer, *Cervus elaphus*). In roe deer, tooth wear that leads the first molar height to decline with increasing age throughout the lifespan allows assessing age of individual roe deer (Veiberg et al., 2007). However, this method is much less accurate than the epigenetic clock (R^2^ = 0.69 vs. 0.93 when a linear regression is used, Figure S2).

Interestingly, the fit of the roe deer epigenetic clock outperforms the few epigenetic clocks previously developed from other mammalian populations. All studies performed so far have investigated the relationship between epigenetic and chronological age through linear regressions, and did not account for nonlinearities. The linear regression provided a better fit in roe deer here (i.e., a coefficient of correlation of .97) than that reported in humpback whales (*Megaptera novaeangliae*, correlation of .89, Polanowski et al., 2014); wood mice, (*Apodemus sylvaticus*, correlation of .92, Little et al., 2020), Bechstein's bat (*Myotis bechsteinii*, correlation of .80, Wright et al., 2018) or yellow baboon (*Papio cynocephalus*, correlation of .78 and .86 for females and males, respectively, Anderson et al., 2021), which to some extent might be due to the broad age range we included in the analysis or to differences in the biological tissue used to extract DNA (e.g., leucocytes vs. wing or ear punches). Overall, the epigenetic clocks used in our study constitute a particularly accurate method for estimating age in roe deer on the basis of leucocyte DNA.

We found that the relationship between epigenetic and chronologic ages was better described by a quadratic than linear model when juveniles were included, but any deviation from a linear model vanished when considering only adults. This discrepancy and the negative second order term of the quadratic model clearly indicate that the relationship between epigenetic and chronological ages is steeper in growing juveniles than in adults. A similar pattern has been reported in humans where the rate of change in DNA methylation profiles (also called “tick rate”) was higher during the developmental period than during adulthood, when a constant tick rate seems to be the rule (Horvath, 2013). The growth period is associated with a high rate of mitotic division and constitutes a particularly demanding life stage in terms of resource allocation in mammals (Reiss, 1989). Although overlooked for a while, the ageing consequences of a fast growth during early life are increasingly investigated (Metcalfe & Monaghan, 2003) and recent evidence suggests that fast growth can shorten lifespan in the long‐run (Kraus et al., 2013; Lee et al., 2013), even though the exact physiological mechanisms underlying this association are likely to be multiple and complex (Metcalfe & Monaghan, 2003; Monaghan & Ozanne, 2018). In roe deer, individuals reach their asymptotic body mass around the age of 4 years in both males and females (Hewison et al., 2011). However, juveniles (i.e., at eight months when individuals are captured for the first time) have already gained about two‐third of their adult body mass (Hewison et al., 2011), with a high amount of individual variation, which makes winter juvenile body mass a reliable measure of growth intensity. We found that the epigenetic acceleration increases with juvenile body mass, suggesting that individuals who allocate substantially in their growth are biologically older than their chronological age indicates, which might contribute to explain why, in roe deer, a fast‐post‐weaning growth is associated with a steeper rate of body mass senescence (Douhard et al., 2017). Such investigations now need to be pursued with a large sample size. Interestingly, a positive association between DNA methylation profiles (as measured with the Horvath pan tissue clock) and height has also been observed among teenagers (Simpkin et al., 2016), which suggests that the discrepancies between biological and chronological age following fast growth might be widespread across mammals and also offer new perspectives for the study of the relationships between growth and ageing.

While mammalian females undeniably live longer than males (Lemaître, Ronget, et al., 2020), studies that have sought to decipher the genetic and physiological correlates of these sex differences in survival have remained rather inconclusive. For instance, other biological markers of ageing such as telomere length or immune performance do not show clear differences between sexes in mammals (Peters et al., 2019; Remot et al., 2020). Yet, gaining a comprehensive view of the physiological and genetic basis of these sex differences in survival prospects is currently a major challenge (Hägg & Jylhävã, 2021). Our analyses of the sex‐specific clock reveal that beyond 7 years of age, females were consistently biologically younger than their chronological age when estimated with the male clock. This suggests that old female roe deer might show a lower degree of biological ageing than males. Yet, we detected no evidence that the epigenetic acceleration was higher in males than in females at any life stage, and further studies controlling for early‐life events (growth trajectories, reproductive allocation) are now required. For sex‐related CpGs, the top enriched data sets were related to X‐linked and Gonosomal inheritance. Other basal sex differences were related to the nervous system (e.g., synapse function), cognition (e.g., intellectual disability, spatial learning), and development (e.g., nervous system, teeth, muscles). We additionally identified two CpGs with basal sex differences that also alter with age in roe deer. The relevance of these CpGs to male excessive in epigenetic age acceleration remained to be tested in experimental studies. Our EWAS was limited in that we only profiled 29,846 highly conserved CpGs that are proximal to 6314 genes. Future EWAS studies could profile the tens of millions of CpGs that are expected to be located in the roe deer genome.

Overall, our study emphasizes that using the epigenetic age acceleration across longitudinal follow‐ups of mammalian populations does constitute to date the most promising approach to (1) estimate accurately the chronological age of individual mammals, (2) assess precisely sex differences in physiological condition and (3) establish reliable predictions in terms of individual trajectories. In the current context of a growing age in human populations associated with pronounced sex differences in the occurrence of age‐associated diseases in the elderly (Austad & Fischer, 2016; Clocchiatti et al., 2016), this research avenue is extremely promising.

## 5 ACKOWLEDGEMENTS

We thank all the OFB staff, in particular Gilles Capron, Stéphane Chabot and Claude Warnant and the field volunteers for organizing the roe deer captures. We are grateful to the four anonymous referees for their helpful comments on this manuscript.

## CONFLICT OF INTEREST

SH is a founder of the nonprofit Epigenetic Clock Development Foundation which plans to license several of his patents from his employer UC Regents. The other authors declare no conflicts of interest.

## AUTHOR CONTRIBUTIONS

Steve Horvath and Jean‐François Lemaître conceived of the study. Steve Horvath and Jean‐François Lemaître wrote the article with inputs from Jean‐Michel Gaillard, Benjamin Rey and Amin Haghani. Jean‐François Lemaître, Benjamin Rey, Jean‐Michel Gaillard, Corinne Régis, Emmanuelle Gilot, François Débias, Jeanne Duhayer, Sylvia Pardonnet and Maryline Pellerin collected data. Jean‐François Lemaître, Steve Horvath, Amin Haghani, Joseph A. Zoller and Caesar Z. Li performed the statistical analyses. All authors reviewed the article.

## Supporting information

Supplementary Material

## Data Availability

The DNA methylation data can be downloaded from Gene Expression Omnibus (GSE184216). In addition, the data used to perform the analyses can be downloaded from dryad (https://datadryad.org/stash/share/aiTxJ‐epCuQWwK4mKroYm7BRzBFCTk30hWHMSNx7PNk ‐ https://doi.org/10.5061/dryad.xd2547dh). Genome annotations of these CpGs and bioinformatics software tools (e.g. normalization) can be found on the Github page of the Mammalian Methylation Consortium https://github.com/shorvath/MammalianMethylationConsortium. The mammalian methylation array (HorvathMammalMethylChip) is distributed by the Epigenetic Clock Development Foundation.

## References

[men13533-bib-0001] Anderson, J. A. , Johnston, R. A. , Lea, A. J. , Campos, F. A. , Voyles, T. N. , Akinyi, M. Y. , & Tung, J. (2021). High social status males experience accelerated epigenetic aging in wild baboons. Elife, 10, e66128. 33821798 10.7554/eLife.66128 PMC8087445

[men13533-bib-0002] Arneson, A. , Haghani, A. , Thompson, M. J. , Pellegrini, M. , Kwon, S. B. , Vu, H. T. , Li, C. Z. , Lu, A. T. , Barnes, B. , Hansen, K. D. , Zhou, W. , Breeze, C.E. , Ernst, J. , & Horvath, S. (2021). A mammalian methylation array for profiling methylation levels at conserved sequences. BioRxiv. 10.1101/2021.01.07.425637 PMC8831611 35145108

[men13533-bib-0003] Austad, S. N. (2010). Methusaleh's zoo: How nature provides us with clues for extending human health span. Journal of Comparative Pathology, 142, S10–S21. 10.1016/j.jcpa.2009.10.024 19962715 PMC3535457

[men13533-bib-0004] Austad, S. N. , & Fischer, K. E. (2016). Sex differences in lifespan. Cell Metabolism, 23(6), 1022–1033. 10.1016/j.cmet.2016.05.019 27304504 PMC4932837

[men13533-bib-0005] Bates, D. , Mächler, M. , Bolker, B. , & Walker, S. (2015). Fitting linear mixed‐effects models using lme4. Journal of Statistical Software, 67(1), 1–48. 10.18637/jss.v067.i01

[men13533-bib-0006] Berger, V. , Lemaître, J.‐F. , Allainé, D. , Gaillard, J.‐M. , & Cohas, A. (2018). Early and adult social environments shape sex‐specific actuarial senescence patterns in a cooperative breeder. The American Naturalist, 192, 525–536. 10.1086/699513 30205028

[men13533-bib-0007] Briga, M. , & Verhulst, S. (2015). What can long‐lived mutants tell us about mechanisms causing aging and lifespan variation in natural environments? Experimental Gerontology, 71, 21–26. 10.1016/j.exger.2015.09.002 26362217

[men13533-bib-0008] Brunet‐Rossinni, A. K. , & Austad, S. N. (2006). Senescence in wild populations of mammals and birds. Handbook of the biology of aging (6th ed.). (pp. 243–266). Elsevier.

[men13533-bib-0009] Burnham, K. P. , & Anderson, D. R. (2003). Model selection and multimodel inference: A practical information‐theoretic approach. Springer Science & Business Media.

[men13533-bib-0010] Chen, B. H. , Marioni, R. E. , Colicino, E. , Peters, M. J. , Ward‐Caviness, C. K. , Tsai, P.‐C. , Roetker, N. S. , Just, A. C. , Demerath, E. W. , Guan, W. , Bressler, J. , Fornage, M. , Studenski, S. , Vandiver, A. R. , Moore, A. Z. , Tanaka, T. , Kiel, D. P. , Liang, L. , Vokonas, P. , … Horvath, S. (2016). DNA methylation‐based measures of biological age: Meta‐analysis predicting time to death. Aging (Albany NY), 8(9), 1844. 10.18632/aging.101020 27690265 PMC5076441

[men13533-bib-0011] Christiansen, L. , Lenart, A. , Tan, Q. , Vaupel, J. W. , Aviv, A. , McGue, M. , & Christensen, K. (2016). DNA methylation age is associated with mortality in a longitudinal Danish twin study. Aging Cell, 15(1), 149–154. 26594032 10.1111/acel.12421 PMC4717264

[men13533-bib-0012] Clocchiatti, A. , Cora, E. , Zhang, Y. , & Dotto, G. P. (2016). Sexual dimorphism in cancer. Nature Reviews Cancer, 16(5), 330. 10.1038/nrc.2016.30 27079803

[men13533-bib-0013] Delorme, D. , Gaillard, J. M. , & Jullien, J. M. (1988). Intérêt de l'étude de la période juvénile pour le suivi de l'évolution d’une population de chevreuils (*Capreolus capreolus*). Gibier Faune Sauvage, 5, 15–26.

[men13533-bib-0014] Douhard, F. , Gaillard, J.‐M. , Pellerin, M. , Jacob, L. , & Lemaître, J.‐F. (2017). The cost of growing large: Costs of post‐weaning growth on body mass senescence in a wild mammal. Oikos, 126(9), 1329–1338. 10.1111/oik.04421

[men13533-bib-0015] Flerov, K. K. (1952). Fauna of USSR, Mammals Vol. 1, No. 2. Musk Deer and Deer. USSR Academia Science.

[men13533-bib-0016] Fletcher, Q. E. , & Selman, C. (2015). Aging in the wild: Insights from free‐living and non‐model organisms. Experimental Gerontology, 71, 1–3. 10.1016/j.exger.2015.09.015 26403678

[men13533-bib-0017] Friedman, J. , Hastie, T. , & Tibshirani, R. (2010). Regularization paths for generalized linear models via coordinate descent. Journal of Statistical Software, 33(1), 1. 10.18637/jss.v033.i01 20808728 PMC2929880

[men13533-bib-0018] Gaillard, J. , Delorme, D. , Boutin, J. M. , Van Laere, G. , Boisaubert, B. , & Pradel, R. (1993). Roe deer survival patterns: A comparative analysis of contrasting populations. Journal of Animal Ecology, 62, 778–791. 10.2307/5396

[men13533-bib-0019] Gaillard, J.‐M. , & Lemaître, J.‐F. (2020). An integrative view of senescence in nature. Functional Ecology, 34(1), 4–16. 10.1111/1365-2435.13506

[men13533-bib-0020] Gaillard, J. M. , Liberg, O. , Andersen, R. , & Hewison, A. J. M. (1998). Population dynamics of roe deer. Scandinavian University Press.

[men13533-bib-0021] Gaillard, J.‐M. , Viallefont, A. , Loison, A. , & Festa‐Bianchet, M. (2004). Assessing senescence patterns in populations of large mammals. Animal Biodiversity and Conservation, 47–58.

[men13533-bib-0022] Hammers, M. , Kingma, S. A. , Spurgin, L. G. , Bebbington, K. , Dugdale, H. L. , Burke, T. , Komdeur, J. , & Richardson, D. S. (2019). Breeders that receive help age more slowly in a cooperatively breeding bird. Nature Communications, 10(1), 1–10. 10.1038/s41467-019-09229-3 PMC6428877 30899016

[men13533-bib-0023] Hewison, A. J. M. , Gaillard, J.‐M. , Delorme, D. , Van Laere, G. , Amblard, T. , & Klein, F. (2011). Reproductive constraints, not environmental conditions, shape the ontogeny of sex‐specific mass–size allometry in roe deer. Oikos, 120(8), 1217–1226. 10.1111/j.1600-0706.2011.19316.x

[men13533-bib-0024] Hewison, A. J. M. , Vincent, J. P. , Angibault, J. M. , Delorme, D. , Laere, G. V. , & Gaillard, J. M. (1999). Tests of estimation of age from tooth wear on roe deer of known age: Variation within and among populations. Canadian Journal of Zoology, 77(1), 58–67. 10.1139/z98-183

[men13533-bib-0025] Holand, H. , Kvalnes, T. , Gamelon, M. , Tufto, J. , Jensen, H. , Pärn, H. , Ringsby, T. H. , & Sæther, B.‐E. (2016). Spatial variation in senescence rates in a bird metapopulation. Oecologia, 181(3), 865–871. 10.1007/s00442-016-3615-4 27033720

[men13533-bib-0026] Horvath, S. (2013). DNA methylation age of human tissues and cell types. Genome Biology, 14(10), 3156. 10.1186/gb-2013-14-10-r115 PMC4015143 24138928

[men13533-bib-0027] Horvath, S. , Erhart, W. , Brosch, M. , Ammerpohl, O. , von Schonfels, W. , Ahrens, M. , Heits, N. , Bell, J. T. , Tsai, P.‐C. , Spector, T. D. , Deloukas, P. , Siebert, R. , Sipos, B. , Becker, T. , Rocken, C. , Schafmayer, C. , & Hampe, J. (2014). Obesity accelerates epigenetic aging of human liver. Proceedings of the National Academy of Sciences, 111(43), 15538–15543. 10.1073/pnas.1412759111 PMC4217403 25313081

[men13533-bib-0028] Horvath, S. , & Levine, A. J. (2015). HIV‐1 infection accelerates age according to the epigenetic clock. The Journal of Infectious Diseases, 212(10), 1563–1573. 10.1093/infdis/jiv277 25969563 PMC4621253

[men13533-bib-0029] Horvath, S. , & Raj, K. (2018). DNA methylation‐based biomarkers and the epigenetic clock theory of ageing. Nature Reviews Genetics, 19(6), 371. 10.1038/s41576-018-0004-3 29643443

[men13533-bib-0030] Hurlbert, S. H. (1984). Pseudoreplication and the design of ecological field experiments. Ecological Monographs, 54(2), 187–211. 10.2307/1942661

[men13533-bib-0031] Ito, T. , Teo, Y. V. , Evans, S. A. , Neretti, N. , & Sedivy, J. M. (2018). Regulation of cellular senescence by polycomb chromatin modifiers through distinct DNA damage‐ and histone methylation‐dependent pathways. Cell Reports, 22(13), 3480–3492. 10.1016/j.celrep.2018.03.002 29590617 PMC5915310

[men13533-bib-0032] Jasinska, A. J. (2020). Resources for functional genomic studies of health and development in nonhuman primates. American Journal of Physical Anthropology, 171, 174–194. 10.1002/ajpa.24051 32221967

[men13533-bib-0033] Jégo, M. , Lemaître, J.‐F. , Bourgoin, G. , Capron, G. , Warnant, C. , Klein, F. , Gilot‐Fromont, E. , & Gaillard, J.‐M. (2014). Haematological parameters do senesce in the wild: Evidence from different populations of a long‐lived mammal. Journal of Evolutionary Biology, 27(12), 2745–2752. 10.1111/jeb.12535 25358546

[men13533-bib-0034] Jung, M. , & Pfeifer, G. P. (2015). Aging and DNA methylation. BMC Biology, 13(1), 1–8. 10.1186/s12915-015-0118-4 25637097 PMC4311512

[men13533-bib-0035] Kennedy, B. K. , Berger, S. L. , Brunet, A. , Campisi, J. , Cuervo, A. M. , Epel, E. S. , Franceschi, C. , Lithgow, G. J. , Morimoto, R. I. , Pessin, J. E. , Rando, T. A. , Richardson, A. , Schadt, E. E. , Wyss‐Coray, T. , & Sierra, F. (2014). Geroscience: Linking aging to chronic disease. Cell, 159(4), 709–713. 10.1016/j.cell.2014.10.039 25417146 PMC4852871

[men13533-bib-0036] Kraus, C. , Pavard, S. , & Promislow, D. E. L. (2013). The size‐life span trade‐off decomposed: Why large dogs die young. The American Naturalist, 181(4), 492–505. 10.1086/669665 23535614

[men13533-bib-0037] Langfelder, P. , & Horvath, S. (2008). WGCNA: An R package for weighted correlation network analysis. BMC Bioinformatics, 9(1), 1–13. 10.1186/1471-2105-9-559 19114008 PMC2631488

[men13533-bib-0038] Languille, S. , Blanc, S. , Blin, O. , Canale, C. I. , Dal‐Pan, A. , Devau, G. , Dhenain, M. , Dorieux, O. , Epelbaum, J. , Gomez, D. , Hardy, I. , Henry, P.‐Y. , Irving, E. A. , Marchal, J. , Mestre‐Francés, N. , Perret, M. , Picq, J.‐L. , Pifferi, F. , Rahman, A. , … Aujard, F. (2012). The grey mouse lemur: A non‐human primate model for ageing studies. Ageing Research Reviews, 11(1), 150–162. 10.1016/j.arr.2011.07.001 21802530

[men13533-bib-0039] Lecomte, V. J. , Sorci, G. , Cornet, S. , Jaeger, A. , Faivre, B. , Arnoux, E. , Gaillard, M. , Trouve, C. , Besson, D. , Chastel, O. , & Weimerskirch, H. (2010). Patterns of aging in the long‐lived wandering albatross. Proceedings of the National Academy of Sciences, 107(14), 6370–6375. 10.1073/pnas.0911181107 PMC2852007 20308547

[men13533-bib-0040] Lee, W.‐S. , Monaghan, P. , & Metcalfe, N. B. (2013). Experimental demonstration of the growth rate–lifespan trade‐off. Proceedings of the Royal Society B, 280(1752), 20122370. 10.1098/rspb.2012.2370 PMC3574304 23235704

[men13533-bib-0041] Lemaître, J.‐F. , & Gaillard, J.‐M. (2017). Reproductive senescence: New perspectives in the wild: Reproductive senescence in the wild. Biological Reviews, 92(4), 2182–2199. 10.1111/brv.12328 28374548

[men13533-bib-0042] Lemaître, J.‐F. , Garratt, M. , & Gaillard, J.‐M. (2020). Going beyond lifespan in comparative biology of aging. Advances in Geriatric Medicine and Research, 2(2), e200011.

[men13533-bib-0043] Lemaître, J.‐F. , Pavard, S. , Giraudeau, M. , Vincze, O. , Jennings, G. , Hamede, R. , Ujvari, B. , & Thomas, F. (2020). Eco‐evolutionary perspectives of the dynamic relationships linking senescence and cancer. Functional Ecology, 34(1), 141–152. 10.1111/1365-2435.13394

[men13533-bib-0044] Lemaître, J.‐F. , Ronget, V. , Tidière, M. , Allainé, D. , Berger, V. , Cohas, A. , Colchero, F. , Conde, D. A. , Garratt, M. , Liker, A. , Marais, G. A. B. , Scheuerlein, A. , Székely, T. , & Gaillard, J.‐M. (2020). Sex differences in adult lifespan and aging rates of mortality across wild mammals. Proceedings of the National Academy of Sciences, 117(15), 8546–8553. 10.1073/pnas.1911999117 PMC7165438 32205429

[men13533-bib-0045] Levine, M. E. , Hosgood, H. D. , Chen, B. , Absher, D. , Assimes, T. , & Horvath, S. (2015). DNA methylation age of blood predicts future onset of lung cancer in the women's health initiative. Aging (Albany NY), 7(9), 690. 10.18632/aging.100809 26411804 PMC4600626

[men13533-bib-0046] Little, T. J. , O'Toole, A. N. , Rambaut, A. , Chandra, T. , Marioni, R. , & Pedersen, A. B. (2020). Methylation‐based age estimation in a wild mouse. BioRxiv. 10.1101/2020.07.16.203687

[men13533-bib-0047] López‐Otín, C. , Blasco, M. A. , Partridge, L. , Serrano, M. , & Kroemer, G. (2013). The hallmarks of aging. Cell, 153(6), 1194–1217. 10.1016/j.cell.2013.05.039 23746838 PMC3836174

[men13533-bib-0048] Lu, A. T. , Fei, Z. , Haghani, A. , Robeck, T. R. , Zoller, J. A. , Li, C. Z. , Zhang, J. , Ablaeva, J. , Adams, D. M. , Almunia, J. , Ardehali, R. , Arneson, A. , Baker, C. S. , Belov, K. , Black, P. , Blumstein, D. T. , Bors, E. K. , Breeze, C. E. , Brooke, R. T. , … Horvath, S. (2021). Universal DNA methylation age across mammalian tissues. BioRxiv. 10.1101/2021.01.18.426733 PMC10501909 37563227

[men13533-bib-0049] MacNulty, D. R. , Smith, D. W. , Vucetich, J. A. , Mech, L. D. , Stahler, D. R. , & Packer, C. (2009). Predatory senescence in ageing wolves. Ecology Letters, 12(12), 1347–1356. 10.1111/j.1461-0248.2009.01385.x 19780789

[men13533-bib-0050] Marais, G. , Gaillard, J.‐M. , Vieira, C. , Plotton, I. , Sanlaville, D. , Gueyffier, F. , & Lemaître, J.‐F. (2018). Sex‐specific differences in aging and longevity: Can sex chromosomes play a role? Biology of Sex Differences, 9, 33. 30016998 10.1186/s13293-018-0181-y PMC6050741

[men13533-bib-0051] Marioni, R. E. , Shah, S. , McRae, A. F. , Chen, B. H. , Colicino, E. , Harris, S. E. , Gibson, J. , Henders, A. K. , Redmond, P. , Cox, S. R. , Pattie, A. , Corley, J. , Murphy, L. , Martin, N. G. , Montgomery, G. W. , Feinberg, A. P. , Fallin, M. D. , Multhaup, M. L. , Jaffe, A. E. , … Deary, I. J. (2015). DNA methylation age of blood predicts all‐cause mortality in later life. Genome Biology, 16(1), 1–12. 10.1186/s13059-015-0584-6 25633388 PMC4350614

[men13533-bib-0052] McLean, C. Y. , Bristor, D. , Hiller, M. , Clarke, S. L. , Schaar, B. T. , Lowe, C. B. , Wenger, A. M. , & Bejerano, G. (2010). GREAT improves functional interpretation of cis‐regulatory regions. Nature Biotechnology, 28(5), 495–501. 10.1038/nbt.1630 PMC4840234 20436461

[men13533-bib-0053] Metcalfe, N. B. , & Monaghan, P. (2003). Growth versus lifespan: Perspectives from evolutionary ecology. Experimental Gerontology, 38(9), 935–940. 10.1016/S0531-5565(03)00159-1 12954479

[men13533-bib-0054] Monaghan, P. , Charmantier, A. , Nussey, D. H. , & Ricklefs, R. E. (2008). The evolutionary ecology of senescence. Functional Ecology, 22(3), 371–378. 10.1111/j.1365-2435.2008.01418.x

[men13533-bib-0055] Monaghan, P. , & Ozanne, S. E. (2018). Somatic growth and telomere dynamics in vertebrates: Relationships, mechanisms and consequences. Philosophical Transactions of the Royal Society B: Biological Sciences, 373(1741), 20160446. 10.1098/rstb.2016.0446 PMC5784066 29335370

[men13533-bib-0056] Nussey, D. H. , Coulson, T. , Delorme, D. , Clutton‐Brock, T. H. , Pemberton, J. M. , Festa‐Bianchet, M. , & Gaillard, J.‐M. (2011). Patterns of body mass senescence and selective disappearance differ among three species of free‐living ungulates. Ecology, 92(10), 1936–1947. 10.1890/11-0308.1 22073785

[men13533-bib-0057] Nussey, D. H. , Froy, H. , Lemaitre, J.‐F. , Gaillard, J.‐M. , & Austad, S. N. (2013). Senescence in natural populations of animals: Widespread evidence and its implications for bio‐gerontology. Ageing Research Reviews, 12(1), 214–225. 10.1016/j.arr.2012.07.004 22884974 PMC4246505

[men13533-bib-0058] Nussey, D. H. , Kruuk, L. E. , Morris, A. , & Clutton‐Brock, T. H. (2007). Environmental conditions in early life influence ageing rates in a wild population of red deer. Current Biology, 17(23), R1000–R1001. 10.1016/j.cub.2007.10.005 18054756

[men13533-bib-0059] Nussey, D. H. , Watt, K. , Pilkington, J. G. , Zamoyska, R. , & McNeilly, T. N. (2012). Age‐related variation in immunity in a wild mammal population: Immune aging in wild sheep. Aging Cell, 11(1), 178–180. 10.1111/j.1474-9726.2011.00771.x 22107028 PMC3397677

[men13533-bib-0060] Paoli‐Iseppi, D. , Deagle, B. E. , McMahon, C. R. , Hindell, M. A. , Dickinson, J. L. , & Jarman, S. N. (2017). Measuring animal age with DNA methylation: From humans to wild animals. Frontiers in Genetics, 8, 106. 10.3389/fgene.2017.00106 28878806 PMC5572392

[men13533-bib-0061] Parrott, B. B. , & Bertucci, E. M. (2019). Epigenetic aging clocks in ecology and evolution. Trends in Ecology & Evolution, 34(9), 767–770. 10.1016/j.tree.2019.06.008 31296344

[men13533-bib-0062] Partridge, L. (2010). The new biology of ageing. Philosophical Transactions of the Royal Society B: Biological Sciences, 365(1537), 147–154. 10.1098/rstb.2009.0222 PMC2842712 20008392

[men13533-bib-0063] Pérez‐Barbería, F. J. , Duff, E. I. , Brewer, M. J. , & Guinness, F. E. (2014). Evaluation of methods to age Scottish red deer: The balance between accuracy and practicality. Journal of Zoology, 294(3), 180–189. 10.1111/jzo.12166

[men13533-bib-0064] Perlman, R. L. (2016). Mouse models of human diseaseAn evolutionary perspective. Evolution, Medicine, and Public Health, 2016(1), 170–176. 27121451 10.1093/emph/eow014 PMC4875775

[men13533-bib-0065] Peters, A. , Delhey, K. , Nakagawa, S. , Aulsebrook, A. , & Verhulst, S. (2019). Immunosenescence in wild animals: Meta‐analysis and outlook. Ecology Letters. 22(10), 1709–1722. 10.1111/ele.13343. 31321874

[men13533-bib-0066] Pettorelli, N. , Gaillard, J.‐M. , Mysterud, A. , Duncan, P. , Chr. Stenseth, N. , Delorme, D. , Van Laere, G. , Toïgo, C. , & Klein, F. (2006). Using a proxy of plant productivity (NDVI) to find key periods for animal performance: The case of roe deer. Oikos, 112(3), 565–572. 10.1111/j.0030-1299.2006.14447.x

[men13533-bib-0067] Polanowski, A. M. , Robbins, J. , Chandler, D. , & Jarman, S. N. (2014). Epigenetic estimation of age in humpback whales. Molecular Ecology Resources, 14(5), 976–987. 10.1111/1755-0998.12247 24606053 PMC4314680

[men13533-bib-0068] Reiss, J. O. (1989). The meaning of developmental time: A metric for comparative embryology. The American Naturalist, 134(2), 170–189. 10.1086/284974

[men13533-bib-0069] Robine, J.‐M. , Herrmann, F. R. , Arai, Y. , Willcox, D. C. , Gondo, Y. , Hirose, N. , Suzuki, M. , & Saito, Y. (2012). Exploring the impact of climate on human longevity. Experimental Gerontology, 47(9), 660–671. 10.1016/j.exger.2012.05.009 22613089

[men13533-bib-0070] Ronget, V. , & Gaillard, J.‐M. (2020). Assessing ageing patterns for comparative analyses of mortality curves: Going beyond the use of maximum longevity. Functional Ecology, 34(1), 65–75. 10.1111/1365-2435.13474

[men13533-bib-0071] Simpkin, A. J. , Hemani, G. , Suderman, M. , Gaunt, T. R. , Lyttleton, O. , Mcardle, W. L. , Ring, S. M. , Sharp, G. C. , Tilling, K. , Horvath, S. , Kunze, S. , Peters, A. , Waldenberger, M. , Ward‐Caviness, C. , Nohr, E. A. , Sørensen, T. I. A. , Relton, C. L. , & Smith, G. D. (2016). Prenatal and early life influences on epigenetic age in children: A study of mother–offspring pairs from two cohort studies. Human Molecular Genetics, 25(1), 191–201. 10.1093/hmg/ddv456 26546615 PMC4690495

[men13533-bib-0072] Snyder‐Mackler, N. , Burger, J. R. , Gaydosh, L. , Belsky, D. W. , Noppert, G. A. , Campos, F. A. , Bartolomucci, A. , Yang, Y. C. , Aiello, A. E. , O'Rand, A. , Harris, K. M. , Shively, C. A. , Alberts, S. C. , & Tung, J. (2020). Social determinants of health and survival in humans and other animals. Science, 368(6493). 10.1126/science.aax9553 PMC7398600 32439765

[men13533-bib-0073] Sugianto, N. A. , Newman, C. , Macdonald, D. W. , & Buesching, C. D. (2020). Reproductive and somatic senescence in the european badger (Meles meles): Evidence from lifetime sex‐steroid profiles. Zoology, 125803. 10.1016/j.zool.2020.125803 32574816

[men13533-bib-0074] Tidière, M. , Badruna, A. , Fouchet, D. , Gaillard, J.‐M. , Lemaître, J.‐F. , & Pontier, D. (2020). Pathogens shape sex differences in mammalian aging. Trends in Parasitology, 36(8), 668–676. 32540194 10.1016/j.pt.2020.05.004 PMC7203054

[men13533-bib-0075] Tower, J. (2017). Sex‐specific gene expression and life span regulation. Trends in Endocrinology & Metabolism, 28(10), 735–747. 10.1016/j.tem.2017.07.002 28780002 PMC5667568

[men13533-bib-0076] Veiberg, V. , Mysterud, A. , Gaillard, J.‐M. , Delorme, D. , Laere, G. V. , & Klein, F. (2007). Bigger teeth for longer life? Longevity and molar height in two roe deer populations. Biology Letters, 3(3), 268–270. 10.1098/rsbl.2006.0610 17311776 PMC2464678

[men13533-bib-0077] Wang, T. , Ma, J. , Hogan, A. N. , Fong, S. , Licon, K. , Tsui, B. , & Bannasch, D. L. (2020). Quantitative translation of dog‐to‐human aging by conserved remodeling of the DNA methylome. Cell Systems. 11(2), 176‐185. 32619550 10.1016/j.cels.2020.06.006 PMC7484147

[men13533-bib-0078] Wilkinson, G. S. , Adams, D. M. , Haghani, A. , Lu, A. T. , Zoller, J. , Breeze, C. E. , & Cooper, L. N. (2021). DNA methylation predicts age and provides insight into exceptional longevity of bats. Nature Communications, 12(1), 1–13. 10.1038/s41467-021-21900-2 PMC7955057 33712580

[men13533-bib-0079] Wright, P. G. R. , Mathews, F. , Schofield, H. , Morris, C. , Burrage, J. , Smith, A. , Dempster, E. L. , & Hamilton, P. B. (2018). Application of a novel molecular method to age free‐living wild Bechstein's bats. Molecular Ecology Resources, 18(6), 1374–1380. 10.1111/1755-0998.12925 29981199

[men13533-bib-0080] Zajitschek, F. , Zajitschek, S. , & Bonduriansky, R. (2020). Senescence in wild insects: Key questions and challenges. Functional Ecology, 34(1), 26–37. 10.1111/1365-2435.13399

[men13533-bib-0081] Zarulli, V. , Jones, J. A. B. , Oksuzyan, A. , Lindahl‐Jacobsen, R. , Christensen, K. , & Vaupel, J. W. (2018). Women live longer than men even during severe famines and epidemics. Proceedings of the National Academy of Sciences, 115(4), E832‐E840. 10.1073/pnas.1701535115 PMC5789901 29311321

[men13533-bib-0082] Zhou, W. , Triche, T. J. Jr , Laird, P. W. , & Shen, H. (2018). SeSAMe: Reducing artifactual detection of DNA methylation by Infinium BeadChips in genomic deletions. Nucleic Acids Research, 46(20), e123. 30085201 10.1093/nar/gky691 PMC6237738

