# Supplementary material for: DNA methylation as a tool to explore ageing in wild roe deer populations
Source: Mol Ecol Resour. 2021 Oct 30;22(3):1002–15. doi: 10.1111/1755-0998.13533 (PMC9297961; doi:10.1111/1755-0998.13533)
Supplement: Supplementary file 1 — Supplementary Material [file MEN-22-1002-s001.docx]

**SUPPLEMENTARY INFORMATION**

**DNA methylation as a tool to explore ageing in wild roe deer populations**

**Jean-François Lemaître^1*^, Benjamin Rey^1^, Jean-Michel Gaillard^1^, Corinne Régis^1^, Emmanuelle Gilot^1,2^, François Débias^1^, Jeanne Duhayer^1^, Sylvia Pardonnet^1^, Maryline Pellerin^3^, Amin Haghani^4^, Joseph A. Zoller^5^, Caesar Z. Li^5^, Steve Horvath^4,5*^**

^1^ Université de Lyon, Université Lyon 1; CNRS, Laboratoire de Biométrie et Biologie Evolutive UMR5558, F-69622 Villeurbanne, France

^2^ Université de Lyon, VetAgro Sup, Marcy-l’Etoile, France

^3^ Office Français de la Biodiversité, Direction de la Recherche et de l’Appui Scientifique, Unité Ongulés Sauvages, F-05000 Gap, France

^4^ Human Genetics, David Geffen School of Medicine, University of California, Los Angeles CA 90095, USA

^5^ Department of Biostatistics, Fielding School of Public Health, University of California, Los Angeles, Los Angeles, California, USA

*Correspondance : [jean-francois.lemaitre@univ-lyon1.fr](mailto:jean-francois.lemaitre@univ-lyon1.fr); [SHorvath@mednet.ucla.edu](mailto:SHorvath@mednet.ucla.edu)

*Running title: Epigenetic clock of roe deer in the wild*

Table S1: Model selection procedure from the set of linear models fitted to test the relationship between DNAm age and chronological age for all individuals in the dataset (A) or for adults only (B). The selected model is highlighted in bold, k is the number of parameters in the model, ΔAIC is the difference in AIC between the candidate model and the selected model. The AIC weight (AICw) is calculated to measure the relative likelihood that a given model is the best among the set of fitted models.

|  |  |  | k | AIC | ∆AIC | AICw |
| --- | --- | --- | --- | --- | --- | --- |
| **(A)** | All individuals (*N*=94) | Constant | 2 | 461.32 | 255.59 | 0.00 |
|  |  | Linear | 3 | 218.00 | 12.26 | 0.00 |
|  |  | **Quadratic** | **4** | **205.73** | **0.00** | **1.00** |
| **(B)** | Individuals older than 1-year-old (*N*=86) | Constant | 2 | 394.75 | 213.81 | 0.00 |
|  |  | **Linear** | **3** | **180.93** | **0.00** | **0.57** |
|  |  | Quadratic | 4 | 181.47 | 0.54 | 0.43 |

Table S2: Model selection procedure from the set of linear models fitted to test the relationship between the epigenetic acceleration and age, sex, population and body mass for adults (A), juveniles (B), Prime-aged individuals (C) and senescent individuals (D). The selected model is highlighted in bold, k is the number of parameters in the model, ΔAIC is the difference in AIC between the candidate model and the selected model. The AIC weight (AICw) is calculated to measure the relative likelihood that a given model is the best among the set of fitted models.

|  |  |  | k | AIC | ∆AIC | AICw |
| --- | --- | --- | --- | --- | --- | --- |
| **(A)** | Individuals older than 1-year-old (*N*=86) | **Constant** | **2** | **184.012** | **1.350** | **0.143** |
|  |  | Body mass | 3 | 185.871 | 3.209 | 0.056 |
|  |  | Sex | 3 | 185.945 | 3.283 | 0.054 |
|  |  | Population | 3 | 184.329 | 1.666 | 0.122 |
|  |  | Body mass + Sex | 4 | 187.858 | 5.195 | 0.021 |
|  |  | Body mass + Population | 4 | 186.155 | 3.492 | 0.049 |
|  |  | Sex + Population | 4 | 186.305 | 3.642 | 0.045 |
|  |  | Body mass + Sex + Population | 5 | 188.015 | 5.352 | 0.019 |
|  |  | Body mass*Sex | 5 | 188.482 | 5.820 | 0.015 |
|  |  | Body mass*Population | 5 | 186.432 | 3.770 | 0.043 |
|  |  | Sex*Population | 5 | 182.662 | 0.000 | 0.280 |
|  |  | Body mass*Sex + Population | 6 | 188.892 | 6.230 | 0.012 |
|  |  | Body mass*Population + Sex | 6 | 188.191 | 5.528 | 0.018 |
|  |  | Sex*Population + Body Mass | 6 | 184.326 | 1.663 | 0.122 |
| **(B)** | Individuals of 1-year-old (*N*=8) | Constant | 2 | 10.374 | 4.942 | 0.064 |
|  |  | Body mass | **3** | **5.432** | **0.000** | **0.753** |
|  |  | Sex | 3 | 12.316 | 6.884 | 0.024 |
|  |  | Population | 3 | 8.535 | 3.103 | 0.160 |
| **(C)** | Individuals between 1-year-old and 8-years-old (*N*=61) | **Constant** | **2** | **106.682** | **1.229** | **0.136** |
|  |  | Body mass | 3 | 108.363 | 2.910 | 0.059 |
|  |  | Sex | 3 | 108.453 | 3.000 | 0.056 |
|  |  | Population | 3 | 105.453 | 0.000 | 0.252 |
|  |  | Body mass + Sex | 4 | 109.761 | 4.308 | 0.029 |
|  |  | Body mass + Population | 4 | 107.293 | 1.840 | 0.101 |
|  |  | Sex + Population | 4 | 107.218 | 1.766 | 0.104 |
|  |  | Body mass + Sex + Population | 5 | 109.180 | 3.728 | 0.039 |
|  |  | Body mass*Sex | 5 | 111.001 | 5.548 | 0.016 |
|  |  | Body mass*Population | 5 | 109.245 | 3.793 | 0.038 |
|  |  | Sex*Population | 5 | 107.316 | 1.863 | 0.099 |
|  |  | Body mass*Sex + Population | 6 | 110.739 | 5.286 | 0.018 |
|  |  | Body mass*Population + Sex | 6 | 111.150 | 5.697 | 0.015 |
|  |  | Sex*Population + Body Mass | 6 | 109.262 | 3.810 | 0.038 |
| **(D)** | Individuals older than 8-years-old (*N*=25) | Constant | **2** | **69.991** | **0.000** | **0.188** |
|  |  | Body mass | 3 | 71.803 | 1.812 | 0.076 |
|  |  | Sex | 3 | 71.868 | 1.877 | 0.073 |
|  |  | Population | 3 | 71.989 | 1.998 | 0.069 |
|  |  | Body mass + Sex | 4 | 73.531 | 3.540 | 0.032 |
|  |  | Body mass + Population | 4 | 73.665 | 3.674 | 0.030 |
|  |  | Sex + Population | 4 | 73.848 | 3.857 | 0.027 |
|  |  | Body mass + Sex + Population | 5 | 75.419 | 5.428 | 0.012 |
|  |  | Body mass*Sex | 5 | 72.905 | 2.914 | 0.044 |
|  |  | Body mass*Population | 5 | 70.349 | 0.358 | 0.157 |
|  |  | Sex*Population | 5 | 70.444 | 0.453 | 0.150 |
|  |  | Body mass*Sex + Population | 6 | 74.242 | 4.251 | 0.022 |
|  |  | Body mass*Population + Sex | 6 | 72.325 | 2.334 | 0.058 |
|  |  | Sex*Population + Body Mass | 6 | 72.225 | 2.234 | 0.061 |

Table S3: Quadratic model describing the relationship between DNAm age and chronological age in roe deer (*N* = 94). Contrary to Table 1, the model was fitted with roe deer identity included as a random effect. Results are qualitatively unchanged (*** *p*<0.001).

|  | Estimate | SE | t |
| --- | --- | --- | --- |
| Intercept | 0.45 | 0.22 | 2.01 |
| Age | 1.11 | 0.08 | 13.87*** |
| Age^2^ | -0.02 | 0.006 | -3.89*** |

Table S4: Parameters of the models including the interaction between chronological age and sex using the female (a) or the male (b) clock; and of the models including the interaction between chronological age and population using the Trois-Fontaines (c) or the Chizé (d) clock. Contrary to Table 2, these models were fitted with the roe deer identity as a random effect. Results are qualitatively unchanged (**p*<0.05, ***p*<0.01, *** *p*<0.001).

| Dependent Variables |  | Estimate | SE | t |
| --- | --- | --- | --- | --- |
| (a) DNAm age [Female epigenetic clock] | Intercept | 1.24 | 0.18 | 6.70*** |
|  | Age | 0.81 | 0.02 | 33.03*** |
|  | Sex | 0.66 | 0.28 | 2.37* |
|  | Age * Sex | -0.08 | 0.04 | -1.80 |
| (b) DNAm age [Male epigenetic clock] | Intercept | 3.46 | 0.19 | 18.34*** |
|  | Age | 0.46 | 0.02 | 18.13*** |
|  | Sex | -1.98 | 0.28 | -6.96*** |
|  | Age * Sex | 0.26 | 0.04 | 6.09*** |

FIGURE S1: Unsupervised hierarchical clustering of the blood samples. We used 1 minus the Pearson correlation matrix as dissimilarity measure in average linkage hierarchical clustering (R function hclust). The clustering branch (first color band) results from a height cut-off of 0.04 (R function cutree). Deer population (second color band) color code Chize (turquoise) and Trois-Fontaines (blue). Hierarchical clustering reveals one severe outlier (third color band). Sex (fourth color band) encodes females (pink) and males (lightblue), grey corresponds to unknown (wrong sex prediction). Age takes white for low values and red for high values.

FIGURE S2: Relationship between molar height (M1, in mm) and age (in years) for roe deer from Chizé and Trois-Fontaines (slope ± se: -0.34 ± 0.02, *N* = 88, R^2^ = 0.69).

**Technical Details surrounding the DNAm age estimator**

**1. Statistical methods used for building the clocks**

The epigenetic clocks were used by employing a single elastic net regression model analysis (R function glmnet). We use used Leave-one-out analysis (LOO) using a single lambda value. We chose the following parameters for the glmnet R function (Alpha: 0.5, CV Fold: 10, Lambda choice for Clock: 1 standard error above minimum CV-MSE).

**2. Covariates and coefficient values of the deer clocks**

1. The roe deer clock for blood samples is based on 61 CpGs whose coefficient values are specified in the column "Coef. RoeDeerBlood". Age transformation=identity, i.e. F(Age)=Age
2. The female roe deer clock for blood samples is based on 41 CpGs whose coefficient values are specified in the column "FemaleRoeDeerBlood". This clock was trained in female samples only. Age transformation=identity, i.e. F(Age)=Age
3. The male roe deer clock for blood samples is based on 32 CpGs whose coefficient values are specified in the column "MaleRoeDeerBlood". This clock was trained in male samples only. Age transformation=identity, i.e. F(Age)=Age

## **3. The DNAm Age estimate is estimated in two steps**

First, one forms a weighted linear combination of the CpGs whose details can be downloaded from Gene Expression Omnibus (GSE184216). We report the probe identifier (cg number) used in the custom Infinium array (HorvathMammalMethylChip40). The weights used in this linear combination are specified in the respective column entitled "Coef.". The formula assumes that the DNA methylation data measure "beta" values but the formula could be adapted to other ways of generating DNA methylation data.
